# Brain trauma impacts retinal processing: photoreceptor pathway interactions in traumatic light sensitivity

**DOI:** 10.1007/s10633-022-09871-1

**Published:** 2022-04-20

**Authors:** Christopher W. Tyler, Lora T. Likova

**Affiliations:** 1 Smith-Kettlewell Eye Research Institute, 2318 Fillmore Street, San Francisco, 94115 USA; 2 Division of Optometry and Vision Science, School of Health Sciences, City University of London, London, UK

**Keywords:** Traumatic Brain Injury, Photosensitivity, ERG, Rods, Cones, Dopamine

## Abstract

**Background:**

Concussion-induced light sensitivity, or traumatic photalgia, is a lifelong debilitating problem for upwards of 50% of mild traumatic brain injury (mTBI) cases, though of unknown etiology. We employed spectral analysis of electroretinographic (ERG) responses to assess retinal changes in mTBI as a function of the degree of photalgia.

**Methods:**

The design was a case–control study of the changes in the ERG waveform as a function of level of light sensitivity in individuals who had suffered incidents of mild traumatic brain injury. The mTBI participants were categorized into non-, mild-, and severe-photalgic groups based on their spectral nociophysical settings. Light-adapted ERG responses were recorded from each eye for 200 ms on–off stimulation of three spectral colors (R:red, G:green, and B:blue) and their sum (W:white) at the highest pain-free intensity level for each participant. The requirement of controls for testing hypersensitive individuals at lower light levels was addressed by recording a full light intensity series in the control group.

**Results:**

Both the b-wave and the photopic negative response (PhNR) were significantly reduced in the non-photalgic mTBI group relative to controls. In the photalgic groups, the main b-wave peak shifted to the timing of the rod b-wave, with reduced amplitude at the timing of the cone response.

**Conclusion:**

These results suggest the interpretation that the primary etiology of the painful light sensitivity in mTBI is release of the rod pathway from cone-mediated inhibition at high light levels, causing overactivation of the rod pathway.

## Introduction

**Fig. 1:**
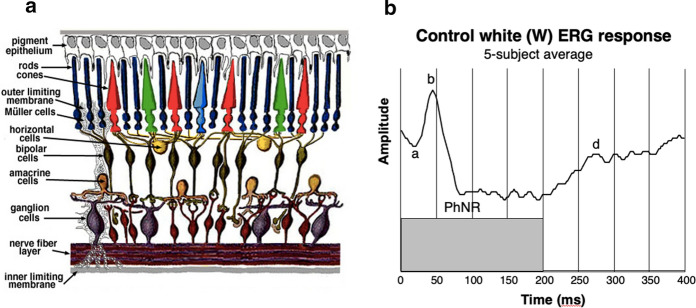
**a** Depiction of the organization of the retinal cell layers. Note the layer of amacrine cells, one type of which gathers the incoming rod responses and relay them to the ganglion-cell layer. ( Source: Webvision: http://webvision.med.utah.edu.) **b** The light-adapted ERG for white light averaged for the control participants to a 200 ms pulse of uniform field stimulation (gray box), showing the a-wave (photoreceptor response), b-wave (bipolar cell response), photopic negative response (PhNR; ganglion cell response), and d-wave (cone off–response). The oscillatory potential is not evident in controls under these light-adapted conditions, and the stimulation is too brief to show the c-wave

For the analysis of retinal dysfunction, the human electroretinogram (ERG) is a highly diagnostic signal of differential function of the retina, of which the main neural organization is diagrammed in Fig. 1a. The typical ERG (Fig. 1b) consists principally of an a-wave (photoreceptors), a b-wave (bipolar cells), a c-wave (pigment epithelium), an oscillatory potential (amacrine cells), and a photopic negative response (PhNR; ganglion cells) [1, 2]. Each component is driven by both the rod and cone photoreceptor types, differentiated by their response dynamics. At higher light levels, rod-driven components are typically slower than cone-driven components, equated for quantum catch. We used the characteristics of these response components to identify retinal mechanism disruptions due to brain trauma.

## ERG Assessment of rod and cone function

The human ERG is often considered a largely rod-dominated response, in view of the much greater number of rods (~ 100 million) than cones (~ 5 million) in the retina [3]. However, the both types of photoreceptors contribute, depending on the light level, and the standard clinical protocol is to use dark-adapted conditions to maximize the rod response and light-adapted ones to maximize the cone response. Use of long-wavelength (red) light pushes the balance of the activation toward the cones and allows both rod and cone b-waves to be seen in the same response [4, 5]. Under light-adapted conditions, the rod b-wave has a peak time about twice as long as that of the cone b-wave, at roughly 60 and 120 ms, respectively ([6]; see its Fig. 12). Since the condition under study is hypersensitivity to light at high intensities, it was important to study the ERG response at high intensities, and to do so across the spectrum in order to differentiate the rod and cone contributions to the response and their interactions in relation to the light hypersensitivity. For this, it was necessary to go beyond the standard red-light dark-adapted protocol, in which the relationship of the rod and cone peaks depends on the duration of dark adaptation [7], and to use a paradigm assessing ERGs at specific light-adaptation levels. Similarly, the photopic negative response (PhNR) protocol specified in [8] is structured to isolate the cone response with the use of a rod-adapting background and therefore does not allow for the assessment of cone/rod interactions under white-light adaptation conditions.

Insight into the nature of photalgia may be sought from the clinical condition of cone dystrophy, which is characterized by a loss of retinal cones and typical reports of severe light-induced pain, or photalgia, at high light intensities [10]. On consideration, however, this photalgic symptom is difficult to explain because it is thought that the switch from rod to cone vision at high light intensities in the healthy retina is due to a compressive saturation of the rod response, leaving the cones as the only photoreceptors still capable of responding. However, if saturation eliminates the rod ERG by preventing the rods from responding to changes in stimulus intensity in the healthy retina, there should equally be no rod ERG response when the cones are removed by the cone dystrophy. The implication of the photalgia in cone dystrophy is therefore that the reduced rod signal at high intensity from the normal retinas must be attributable to some form of cone-rod suppression mechanism that is released when the cones are lost in cone dystrophy.

Moreover, there has been a series of reports of supernormal rod ERG b-wave amplitudes in cases of cone dystrophy when measured at high intensities [11–20], although no clear mechanism for the supernormal rod ERG has been established. Assessment of the visual function in such cases behaviorally shows that not only is rod visual detection not supernormal in these cone dystrophy cases, but it is in fact degraded to a level comparable to the reduction in cone function [21]. Thus, the supernormality of the cone dystrophy ERG seems to be more a factor of signal strength than of the signal-to-noise level controlling detectability under rod-isolation conditions.

The retina is known to be susceptible to damage from mTBI in rodents, notably with cell loss in the inner plexiform layer and with retinal nerve fiber laying thinning [22, 23]. The latter study also reported a reduction in the PhNR in the full-field ERG of the mTBI mice. Corresponding visual field losses from mTBI have also been reported in humans [24, 25].

As many as 50% of individuals subjected to mild traumatic brain injury (mTBI) experience abnormal light sensitivity (photalgia) at high light levels [26]. To study the relative involvement of the retinal rod and cone pathways in such light sensitivity (photalgia) at high light levels, we measured ERG responses across the spectrum in groups of participants classified as non-mTBI Controls, and mTBI with no, mild, or severe degrees of photalgia (***non-Ph, mild-Ph***, and ***severe-Ph*** mTBI, respectively).

## Methods

### Recruitment

The 20 mTBI study participants, aged 54.5 ± 3.4 years (60% male), were recruited from a non-academic population via a social media website analysis if they had a letter acuity of 20/40 or better in both eyes. The mTBI group had closed-head trauma at least three weeks prior to the ERG recording with either report of loss of consciousness (LOC) for some duration, or loss of memory of the traumatic event for the two cases with no LOC. The seven aged-matched controls, aged 47.7 ± 8.2 years old (57% male), had no reportable head trauma events. All procedures adhered to the Declaration of Helsinki and was approved by the SKERI institutional review board; informed consent was obtained from all participants, none of whom withdrew from the study. Participant characteristics are tabulated in Table 1.

**Table 1:** Participant demographics and visual characteristics

Condition	Partice	Gender	Age range	Photalgia status	Number of conc	Hours of LOC	Years since last	Reported deficits	Medical problems	Memory problems
mTBI	MRGC005	M	60 s	Non-Ph	1	> 0	40.0			0
	MRGC006	F	20 s	Non-Ph	1	0.1	5.0			0
	MRGC007	M	60 s	Non-Ph	1	0.08	0.1		Heart	2
	MRGC011	F	20 s	Non-Ph	1	0.5	5.0	Headache		0
	MRGC012	M	40 s	Non-Ph	1	168	4.0			2
	MRGC013	M	50 s	Non-Ph	1	72	40.0	Sleep dist	Asthma	0
	MRGC016	M	60 s	Non-Ph	3	0.05	14.0	Sensitivity		0
	MRGC001	M	60 s	Mild Ph	1	0.05	0.1	Headache		2
	MRGC002	M	80 s	Mild Ph	1		3.0	Balance	Stroke	2
	MRGC003	M	50 s	Mild Ph	1	1	6.0	Sleep dist		0
	MRGC009	M	50 s	Mild Ph	2	0.5	0.9	Sleep dist		0
	MRGC010	M	40 s	Mild-Ph	1	0.8	2.0			2
	MRGC015	F	60 s	Mild Ph	1	0.13	5.0	Headache	Herbs	0
	MRGC017	F	30 s	Mild Ph	1	0.02	9.0	Sleep dist		2
	MRGC018	F	50 s	Mild Ph	2	0.03	8.0	Headache		0
	MRGC020	F	40 s	Mild Ph	2	0.08	44.0		Headache	2
	MRGC004	M	80 s	Severe-Ph	2	20	4.0	Balance	Cataract	2
	MRGC008	F	60 s	Severe Ph	1		0.3			2
	MRGC014	M	50 s	Severe Ph	5	0.5	19.0	Balance	HIV	0
	MRGC019	F	40 s	Severe Ph	2	4	12.0	Sleep dist	Anxiety	0
Controls	MRGC021	M	60 s	Non-Ph						0
	MRGC022	M	30 s	Non-Ph						0
	MRGC023	F	50 s	Non-Ph						0
	MRGC024	M	70 s	Non-Ph						0
	MRGC025	M	30 s	Non-Ph						0
	MRGC026	F	30 s	Non-Ph						0
	MRGC027	F	20 s	Non-Ph						0

*M* Male, *F* Female, Partic. # Participant number, ph. photalgia, conc. Concussions, *LOC* Loss of consciousness, > 0 some LOC but duration not recalled, Sleep dist. Sleep disturbance, *HIV* Human immunodeficiency virus infection

The individuals designated as ***non-Ph*** mTBI group if they reported no discomfort on viewing the full-field flickering stimuli at their maximum intensities, and to one of the ***Ph*** groups if they required a reduced intensity of any of the stimuli for the recording procedures.

### Stimuli

Full-field ERG recording was performed with a high-intensity CRT monitor display, expanded to the full field of view with a mirror-reflective hood extending to the face. Its narrowband phosphors provided chromatic stimuli with peak wavelengths of 610 nm (R), 540 nm (G), and 480 nm (B), together with a broadband stimulus (W = R + G + B). Each color was alternated with darkness at 2.5 Hz (200 ms on/ 200 ms off) for 60 s (150 cycles), consistent with the ISCEV extended protocol for the photopic on–off ERG [9]. This ISCEV protocol does not provide a definitive specification for a background light, other than implying that it should be chosen to provide for selective stimulation of the photoreceptor type(s) of concern in the study. Our concern is to assess the relative responses of rods and cones at each wavelength, for which purpose the optimal strategy is to use no background light for the on–off stimulation, allowing all photoreceptor types to respond without impediment.

Because this was a study of photalgia, and the usual approach of recording with dilated pupils at maximum stimulus intensity would have caused undue hardship, we adopted the alternative approach of stimulating with natural pupils at the highest intensity that was comfortable for the participant. Our full-field stimulation hood did not allow visual access to the face for measurement of the pupil sizes during stimulation. The retinal illuminances (Table 2) were therefore calculated through pupil sizes according to the unified formula of [27].

**Table 2:**
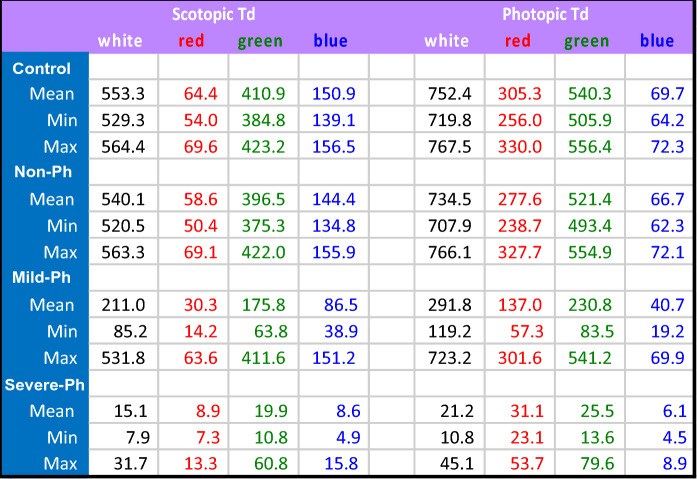
Intensity parameters for each color condition for each mTBI/photalgia group

We note that the literature on sustained pupil size changes in mTBI, even in the subacute phase, is inconclusive. Some subacute studies show increases (e.g., [28]), some show decreases (e.g., [29, 30]), and some show no significant changes (e.g., [26]). One study of chronic mTBI found a significant restriction of about 11% in pupil diameter [31]. Thus, any small changes in pupil diameter are unlikely to account for large changes in chronic ERG waveform reported in our study, which would require a log unit change in retinal illuminance to account for the present results.

### ERG recording

ERGs were recorded by means of the EGI Geodesic Netstation high-density, whole-head recording system, which incorporates 128 skin electrodes distributed around the head and face. (This novel recording strategy was adopted in view of the sensitivity of many photalgic participants to light, allowing us to obtain ERGs within the informed consent guidelines without the use of corneal electrodes.) The ERG signal for each eye was derived from the differential signal between the electrode located immediately below the eye and the one at the temple adjacent to the eye, similar to the standard skin electrode configuration [32]. Recording bandwidth was 0.01–250 Hz with an amplifier gain of 20,000.

A control intensity series with the white light (W) stimulus was run on the seven control participants matched in age to the mTBI group and gender distribution, as specified in Table 1, to provide a baseline for comparison with the mTBI participant group tested at their maximum tolerable intensities. The control intensities were set at the mean white level specified in Table 2 and five successive levels decreasing in factors of 2 (248, 124, 62, 31, 15.5 cd/m^2^; with calculated retinal illuminances as in Table 2).

## Results

### Qualitative description

**Fig. 2:**
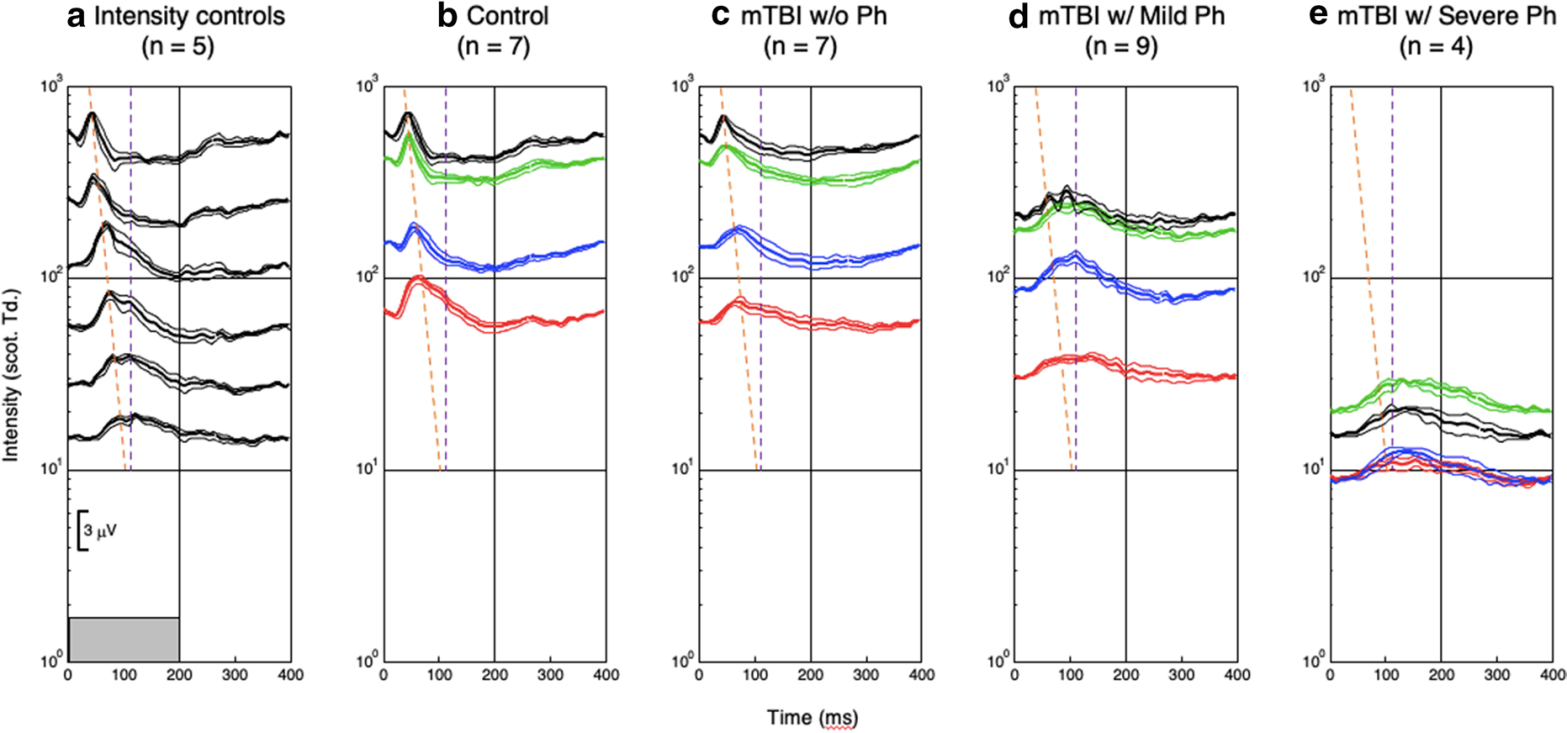
Group-averaged ERG waveforms to the 200 ms stimulus pulses in the ***control*** intensity series (**a**), ***control*** color set (**b**), and ***non*** (**c**), ***mild*** (**d**), and ***severe*** (**e**) ***Ph*** groups. Each panel shows the time courses of the average ERG responses to the R, G, B and W color fields (red, green, blue and black traces) for the designated group, vertically displaced at time zero in terms of scotopic retinal illuminance (scot. Td). Thin curves show ± 1 SEM ranges around each average waveform. Orange and violet dashed lines are a fit to the initial b-wave peak times for the ***control*** intensity series and a line of a fixed delay of 110 ms representing the peak of the rod response, respectively. Note that the ERG waveforms brain trauma without photalgia (***non-Ph*** group) are substantially altered at the highest intensities (**c**), losing the photopic negative response following the positive peak (in **b**), but with little change in the peak times. On the other hand, the ***mild-Ph*** group shows a marked slowing of the peak response (**d**), despite the illuminances remaining in a range overlapping with the ***control*** intensity series. The ***severe-Ph*** group (**e**) shows further slowing attributable to the low illuminances. These qualitative observations are supported by the statistical analyses

High-quality ERG responses were obtained for full-field stimulation of whole-field chromatic stimuli at 2.5 Hz On–Off stimulation, with peak wavelengths of R (610 nm), G (540 nm), B (480 nm), and a W (R + G + B) stimulus with the mean intensity adjusted as necessary for the comfort of the photalgic participants. (see Table 2 for the resultant intensity ranges of each group). A control W intensity series (Fig. 2a) provided waveform comparisons with those participants with reduced intensity settings. Note that the waveforms for all conditions are displaced vertically at their 0 ms level on the y-axis, with the response amplitudes referred to the millivolt calibration bar in the inset. The following qualitative description focuses on waveform features that differ by greater than 3 standard errors of the means (SEM).

At the maximum intensity, the average control W waveform shows the classic a-b-PhNR morphology for the W stimuli (see Fig. 1b). At the lower intensities in Fig. 2a, it is notable that the average b-wave peak is delayed and appears to separate into two peaks, interpretable as separate cone (earlier peak) and rod (later peak) contributions to the waveforms [5]. The color ERG responses for the control group (Fig. 2b) again showed the classic a-b-PhNR morphology as for the W stimuli (compare Fig. 1b), with a similar structure for the G response, but the B and R responses have lost much of the PhNR dip and the b-wave peak time is slightly delayed.

The responses of the ***non-Ph*** mTBI group (Fig. 2c) show a notable reduction in amplitude relative to the full-intensity ***control*** group (Fig. 2b). They also show a marked loss in the negativity of the PhNR immediately following the primary peak. Beyond these changes, the ***non-Ph*** mTBI group (Fig. 2c) shows little difference in peak times from the controls except for the B response, which is noticeably slowed. The ***mild-Ph*** group (Fig. 2d) shows further slowing relative to the ***control*** group, when compared at comparable baseline levels (indexed by the height in the y-axis). Two further aspects of the ***mild-Ph*** mTBI group are worthy of note. One is that the W responses show evidence for the well-known oscillatory potential of the ERG (Fig. 2d), which is a primarily rod function found at high intensities [33]), and are not seen in the rapid peak time (cone) ***control*** responses (Fig. 2b). The other is that the R responses show a hint of an earlier peak at the time predicted for the cone responses, providing some information about the level to which the cone responses have been reduced in the ***mild-Ph*** group. The ERG responses for the ***severe-Ph*** group (Fig. 2e) continue the same trends as for the ***mild-Ph*** group. Finally, the responses are further slowed, but no more than expected from the reduced illuminance level set by these participants.

**Fig. 3:**
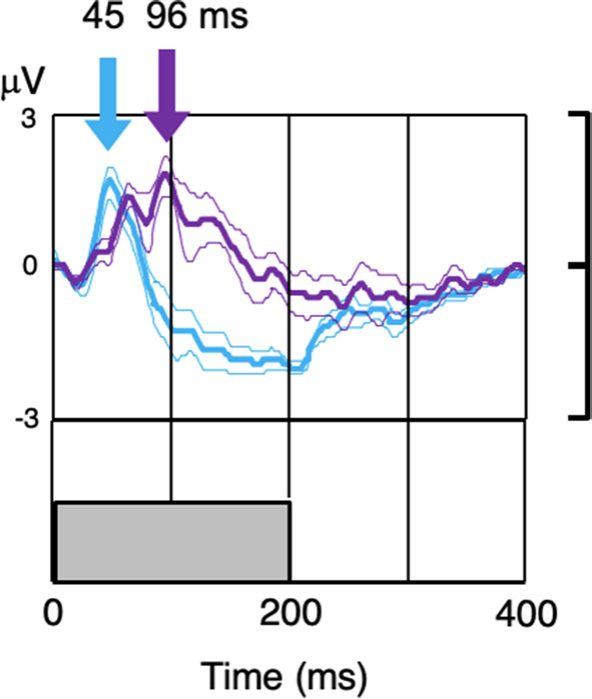
Average ERG waveform comparison for the ***mild-Ph*** group (violet curve) with the nearest intensity from the ***control*** group (cyan curve). Thin curves are ± 1 SEM error ranges. Portions of the curve separated vertically by more than 3 SEM imply significant shape differences (e.g., at about 50 ms and from about 90 to 210 ms)

To make the point directly, Fig. 3 compares the average ERG response for the ***control*** group, which is compared with that in the ***mild-Ph*** mTBI group at the closest available intensity (which was slightly lower for the ***control*** group, 212 vs 245 scotopic Td). It can be seen that the waveform is dramatically slowed in the patient group, with time to peak of 96 vs 45 ms, despite the luminance being equated (or actually slightly higher) for the patient group. Amplitudes are comparable at about 2 μV.

### Statistical analysis

To quantify the results described in Fig. 2, they were analyzed in three ways. The data analysis is focused the features identified in the control intensity series of Fig. 2a to define a set of fiducial timepoints at which to assess the waveform changes in terms of standard ERG components. These timepoints (with their target ERG components) are 20 ms (a-wave), 40 ms (cone b-wave), 80 ms (PhNR, rod b-wave), 120 ms (i-wave), 140 ms (c-wave) and 200 ms (late baseline level). Note that the ERG component labels are given here as an interpretive guide based on the present dataset, not necessarily adhering to their formal definitions, which are not specified for these recording conditions.

To account for the luminance differences at each photalgia level, the white ***control*** intensity series provided the comparison waveforms in the intensity range of the photalgia setting for each participant, derived according to the slope functions shown in Fig. 2a. The statistics were then run on the difference between each mTBI/photalgia waveform and the average ***control*** W waveform at the intensity closest to that of the mTBI/photalgia waveform (i.e., taking the photalgic intensity setting into account).

A three-way analysis of variance (ANOVA) was run to compare the patient and control ERG signals on the six timepoints (20, 40, 80, 120, 140, and 200 ms) by four color conditions (white, red, green, and blue) by four photalgia groups (***control, non-Ph, mild-Ph***, and ***severe-Ph***). This ANOVA (Table 3) shows highly significant main effects of **timepoint**, **color condition**, and **mTBI/photalgia group**. It also shows highly significant interactions between some pairs of these variables, the **timepoint-by-color-condition** interaction and the **color-condition-by-TBI/photalgia group** interaction, though not the **timepoint-by-TBI/photalgia group** or the three-way interaction. This analysis thus reveals strong effects of each of the principal ERG parameters for the assessment of photalgia.

**Table 3:**
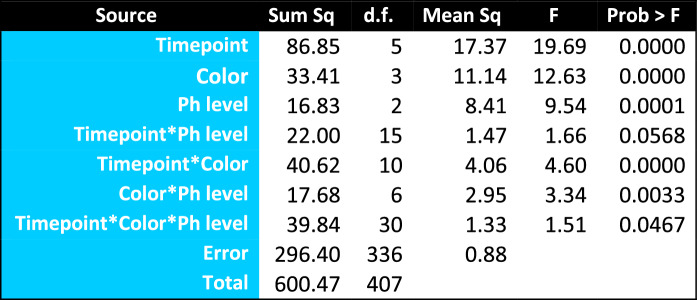
Three-way ANOVA

However, the three-way analysis does not take into account the variation in effective luminance of the individual colors making up equal-energy white (as shown by their relative placement on the intensity ordinate in Fig. 2. We therefore ran separate two-way ANOVAs for the each of the four-color conditions (R, G, B, and W) for the unbiased difference functions of the mTBI/photalgia participants relative to the respective mean ***control*** waveforms (Table 4), with the following results:

1. Significant main effect of ***timepoint*** for conditions G, B and W: G timepoint (F(5) = 9.36 *p* < 0.0000); B timepoint (F(5) = 6.70 *p* < 0.0000); W timepoint (F(5) = 6.16, *p* < 0.0001);
2. Significant main effect of ***mTBI/photalgia*** group (***Ph*** level) for condition B (F(2) = 8.30, *p* = 0.0006), and close to the significance level for conditions G and W;
3. Significant interactions between ***timepoint*** and ***mTBI/photalgia*** group for conditions G and W; G timepoint x photalgia level interaction (F(10) = 3.74, *p* < 0.0004); W ***timepoint*** x ***Ph*** level interaction (F(10) = 2.59, *p* < 0.0087).

**Table 4:**
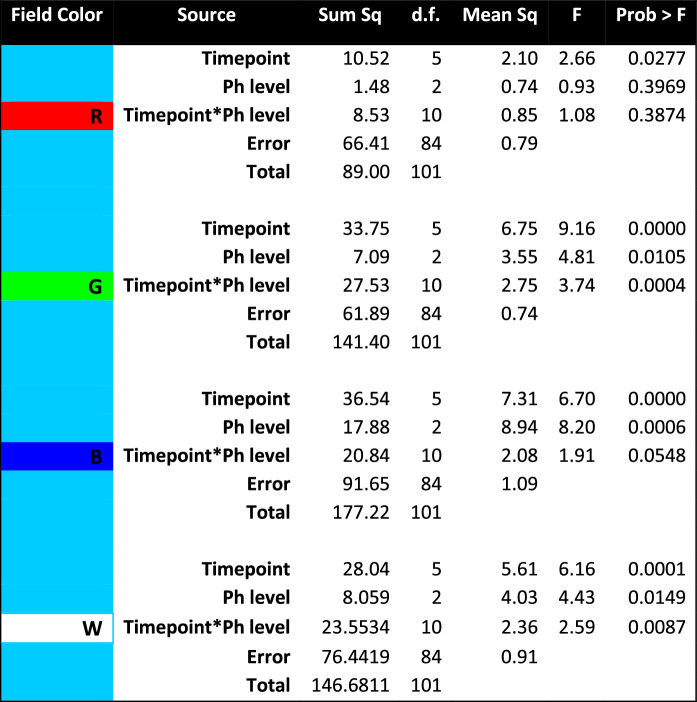
Two-way ANOVAs for each color condition

## Discussion

This study focused on the retinal sequelae to mTBI in relation to the reported symptom of light-induced pain, or photalgia. Surprisingly, the light-adapted ERG responses revealed a significant pattern of retinal losses in the brain trauma groups as function of degree of photalgia. In order to interpret this pattern, we need to analyze the behavior of the ***control*** responses as a function of mean intensity, as shown in Fig. 2a. At the highest intensities, the ***control*** group showed the typical ERG pattern of a small a-wave, a purely photopic b-wave and a typical PhNR, but at moderate intensities the PhNR dip was progressively reduced and replaced by the suggestion of a second peak, which is interpretable as the rod b-wave beginning to emerge and progressively override the PhNR. This same of response pattern was replicated in the color responses for the ***control*** group (Fig. 2b), with respect to their relative intensities.

The brain trauma ***non-Ph*** mTBI group without photalgia (Fig. 2c) showed a similar loss of the PhNR, but now occurring at the highest intensities. This pattern is repeated in the ***mild-Ph*** group (Fig. 2d), though they were tested at the somewhat reduced intensity settings required by their photalgia. The main peak has now shifted to the timing of the rod b-wave (violet dashed line), with a residual peak evident at the timing of the cone response (orange dashed line).

The usual interpretation of such a delayed response peak is that it is driven by the rod (scotopic) system rather than the cone (photopic) system of the retina [3], which would imply that the rod b-wave amplitude is particularly increased in photalgia relative to the cone b-wave amplitude. The inference to be drawn from this relationship is therefore that, rather than being saturated at maximum response at everyday photopic illumination levels, the rod pathway is inhibited by the activity in the cone pathway [34] and is hence inactive for shorter light wavelengths in the daylight. Apparently the longer light wavelengths are, perhaps paradoxically, unable to activate this inhibitory pathway, with the consequence that both rod and cone signals are recordable under these conditions, as historically reported [4–6]. The data suggest, however, that the effect of the mTBI is to reduce the cone response (as evidenced by the lack of a peak at the cone latency predicted from the ***control*** group, as shown in Fig. 3) and to release the rod response from cone-generated inhibition, allowing it to be revealed at these high light levels where the rod pathway is normally inactive. On this interpretation, photalgia at photopic illumination levels may result from overactivity of the rod pathway due to the loss of its normal inhibition by the cone system.

It is possible to propose, given the indirect measure of pupil diameter imposed by our full-field stimulation setup, that the effect of the mTBI was to affect the pupil control mechanism and constrict the pupils sufficiently to cause the ERG to exhibit the rodlike properties seen in Figs. 2 and 3. However, this effect is implausible as it would require a reduction in intensity of about a factor of 10, which is at the limit of the physiological range for a 4 mm pupil.

### Biochemical effects of mild traumatic brain Injury

There is a well-established literature on the disruption of catecholamines, specifically dopamine, in mTBI. Jenkins et al. [35] used 123I-Ioflupane SPECT to find that moderate-severe mTBI causes significant depletions of dopamine levels in the human caudate nucleus and reduced substantia nigra volume. These brain effects have also been shown to affect systemic dopamine [36] and may well constitute a pathway for the downregulation of dopaminergic neurons in the retina, such as the inhibitory dopaminergic amacrine cells, which have a key role in the mutual inhibitory interactions between the rod and cone pathways in the retina [34]. In support of the dopamine involvement following the stress of brain trauma, Yehuda et al. [37] found a significant association between urinary dopamine excretion levels in post-traumatic stress disorder (PTSD, which has high co-morbidity with mTBI) and symptom severity.

### Reciprocal dopamine-mediated cone-rod suppression

The inferred mechanism of cone-rod suppression could be mediated by the retinal dopamine transmitter. Dopamine is released by a unique set of amacrine cells in the inner plexiform layer of the retina and activates D1 and D2 dopamine receptors distributed throughout the retina [34]. These **AII** amacrine cells are the primary pathway for the rod photoreceptor signals to the retinal ganglion cell output from the retina. Moreover, there is mutual dopamine-mediated cone-rod inhibition in the retina, facilitating the switch from rod to cone function at mesopic light levels, and providing for rod suppression at high light levels [34]. These dopamine effects are identifiable in the human pattern ERG, which shows a dip in the spatial tuning function in Parkinsonian patients with reduced dopamine function [38]. This characteristic pattern-ERG dip showed recovery following treatment with L-dopa, the ingestible precursor to dopamine [39], and it is notable that common forms of Parkinson’s are strongly associated with photophobia/photalgia [39, 40]. Overall, these findings are consistent with the concept of dopamine acting as a chemical messenger for rod suppression by cone signals under everyday light-adaptation conditions.

## Conclusion

Taken as a whole, the present results form an unexpected constellation of retinal effects of brain trauma. The shift from a photopic to a scotopic b-wave in photalgic brain trauma can be interpreted to imply that dopamine-mediated cone-to-rod suppression operating under normal conditions at high light levels is blocked by the effects of the brain trauma in photalgic cases. Thus, the effect of the brain trauma may be to release the rod pathway from the regular inhibition, causing its overactivation at moderate light levels. This concept of overactivation of the rod pathway under light-adapted conditions provides a natural explanation for the otherwise unexplained light sensitivity symptoms in the photalgia groups.

## Data Availability

The raw data for this study will be made available on request to the first author.

## Declarations

### Statement on the welfare of animals

This article does not contain any studies with animals performed by any of the authors.
